# Peretinoin, an Acyclic Retinoid, for the Secondary Prevention of Hepatocellular Carcinoma

**DOI:** 10.3390/molecules26020295

**Published:** 2021-01-08

**Authors:** Hyun Young Woo, So Young Yoo, Jeong Heo

**Affiliations:** 1 Department of Internal Medicine, College of Medicine, Pusan National University and Medical Research Institute, Pusan National University Hospital, Busan 49241, Korea; who54@hanmail.net; 2 BIO-IT Foundry Technology Institute, Pusan National University, Busan 46241, Korea

**Keywords:** chemoprevention, hepatocellular carcinoma, peretinoin

## Abstract

The high rates of hepatocellular carcinoma (HCC) recurrence after initially successful curative therapy emphasize ongoing unmet needs to prevent or reduce HCC recurrence. Retinoid acid (RA), a metabolite of vitamin A and its related analogues (termed retinoids) has been suggested as a promising chemotherapeutic agent in cancer treatment. The synthetic oral retinoid peretinoin is the only agent for the secondary chemoprevention of HCC after curative therapy that is currently well applied into clinical development. Here we present an updated summary of the molecular pathogenesis of HCC and of preclinical and clinical findings with peretinoin, including its clinical characteristics, safety and tolerability profile and future perspectives for clinical use.

## 1. Introduction

Liver cancer is the fifth most common neoplasm in men and the seventh most common neoplasm in women worldwide [1,2]. The incidence and mortality rates of liver cancer are particularly high in Asia, with over 75% of patients with liver cancer being from Asian countries [1,3]. Hepatocellular carcinoma (HCC) is the most common type of liver cancer. The prognosis of patients with HCC has improved recently owing to developments in various therapeutic methods, including surgical resection, ablation therapy [mostly radiofrequency ablation (RFA), percutaneous-ethanol-injection (PEI)], transarterial chemoembolization (TACE), and molecularly targeted agents such as sorafenib, lenvatinib, and regorafenib, liver transplantation [4,5,6,7,8,9]. Despite successful treatment, however, HCC has a high risk of recurrence. The mode of recurrence is identical in patients with HCC caused by hepatitis B virus (HBV) and hepatitis C virus (HCV) and is thought to be due to manifestations of intrahepatic metastases and metachronous multicentric carcinogenesis. Moreover, although it is difficult to determine the mode of recurrence of individual lesions, the timing of recurrence is believed to differ. For example, recurrence from intrahepatic metastases is predominant within 2 years after radical treatment of the primary tumor, whereas metachronous multicentric recurrence occurs predominantly after 2 years [10,11]. Most patients ultimately die of HCC, due to the occurrence of non-responding lesions, such as intraportal tumor thrombi, diffuse multiple cancers, and distant metastases.

In addition to early detection and early treatment, the prognosis of patients with HCC may be improved by aggressively suppressing HCC recurrence. Antiviral therapy, mainly with nucleic acid analogs and Direct acting antivirals (DAAs), may inhibit the recurrence of HBV- and HCV-positive HCC after radical treatment of patients [12,13]. However, there is no established non-viral strategy to prevent recurrence of virus associated HCC after curative therapy. The incidence of HCC in patients with non-alcoholic fatty liver disease (NAFLD) and metabolic syndrome has recently increased [14]. Because HCC can develop in patients with NAFLD, even in the absence of established cirrhosis, to prevent recurrence of non-virus associated HCC after curative therapy, there is an urgent unmet need for secondary chemoprevention of HCC recurrence after curative therapy. This review investigated the current status and prospects of peretinoin, an acyclic retinoid (ACR) compound developed for secondary prevention of HCC.

## 2. Peretinoin

Peretinoin is a synthetic polyprenoic acid that binds to cellular retinoic acid-binding protein [15] and has retinoid-like properties. Initially, it was developed under the name E-5166 (Eisai Co., Ltd.) to treat dermal diseases. A randomized placebo controlled study showed that oral administration of peretinoin (600 mg/day, twice daily) for one year to patients who had undergone curative resection or percutaneous ethanol injection therapy for viral and non-viral HCC showed good tolerability, inhibited HCC recurrence, and improved patient survival rate [16,17]. Based on these promising results, peretinoin was developed under the name NIK 333 (Kowa Company, Ltd.) in 1997, clinical studies were started in February 2012, and the name of the compound was changed to K-333. The mechanism of action of peretinoin includes transcriptional activation via the retinoic acid receptor (RAR) and retinoid X receptor (RXR), promoting, along with other transcriptional complexes, the transcription of target genes. Peretinoin was found to modulate genes involved in the regulation of cellular proliferation, cellular differentiation and apoptosis in HCC cells [18,19,20]. Several pharmacologic studies have indicated that peretinoin inhibits the recurrence of HCC by inhibiting carcinogenesis of precancerous lesions in the liver and/or by inhibiting the growth of subclinical cancers [19,21].

Peretinoin is currently undergoing extensive pre-registration phase III placebo-controlled trials in Asia to evaluate its efficacy and safety in subjects with virus-associated HCC following complete tumor removal (Table 1).

### 2.1. Chemistry

**Scheme 1:**
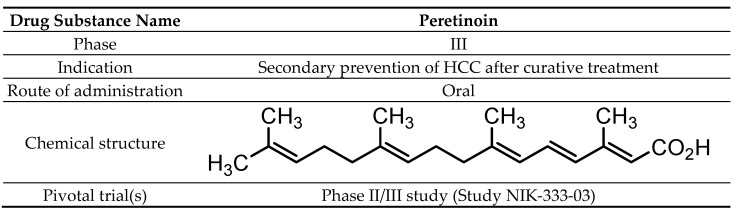
Drug substance summary.

The chemical name of peretinoin is (2*E*,4*E*,6*E*,10*E*)-3,7,11,15-tetramethylhexadeca-2,4,6,10,14-pentaenoic acid (Scheme 1). Peretinoin consists of crystals or crystalline powder, pale yellow to yellow in color, with a molecular formula of C_20_H_30_O_2_ and a molecular weight of 302.45 g/mol. Peretinoin was highly soluble in dimethylsulfoxide, soluble in ethanol (99.5%) and acetone, sparingly soluble in methanol, slightly soluble in acetonitrile and hexane, and practically insoluble in water.

### 2.2. Pharmacodynamics

Peretinoin was found to activate transcription through retinoic acid receptor (RAR) and retinoid X receptor (RXR), and could activate the differentiation of human acute promyelocytic leukemia in HL-60 cells [22,23]. Peretinoin significantly inhibited hepatocarcinogenesis in rat chemical carcinogenesis models using 3′-methyl-4-(dimethylamino)azobenzene (3′-MeDAB) and diethylnitrosamine (DEN) [24,25], suggesting that peretinoin was also involved in suppressing hepatocarcinogenesis. Furthermore, peretinoin was reported to reduce the numbers of oval cells with stem cell-like properties that appear during early stages of 3′-MeDAB-induced hepatocarcinogenesis in rats [26,27]. All-trans-RA was found to induce the differentiation of hepatic precursor cells derived from mouse fetal liver cells [28], and peretinoin is thought to act similarly. Peretinoin treatment also prevented obesity-related liver carcinogenesis and attenuated liver steatosis and inflammation [29], thereby being expected to reduce HCC incidence in patients with liver cirrhosis. These results suggest that, due to its retinoid-dependent and -independent effects, peretinoin prevents the recurrence of HCC by inhibiting oncogenesis of precancerous lesions in the liver and/or by inhibiting the growth of occult hepatic cancer. In addition to preclinical study, the effect of peretinoin in human was investigated through clinical study (NIK-333-02) [18]. In this study, the change of gene expression profile after 8 weeks of peretinoin treatment was examined in liver biopsy for patients who had received curative treatment for HCC. The levels of expression of genes encoding interferon, tumor suppressors, negative regulators of Wnt and insulin-like growth factor (IGF) signaling, hepatocyte differentiation and retinoid-induced genes were higher after 8 weeks of peretinoin treatment than before treatment. In contrast, genes related to mammalian target of rapamycin (mTOR), tumor progression, the cell cycle and metastasis/angiogenesis were downregulated. Through retinoid target gene, peretinoin inhibit HCC proliferation, suppress tumor growth or induce tumor apoptosis [30,31,32,33].

**Figure 1:**
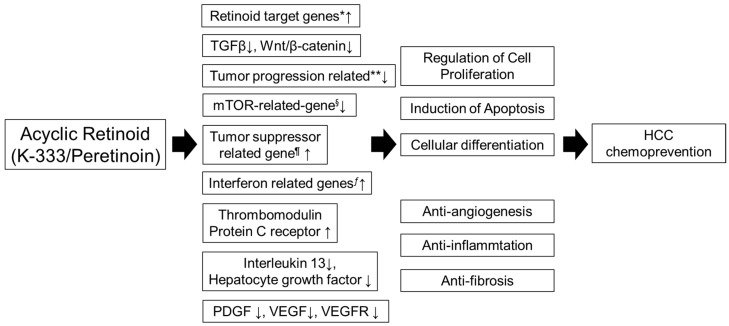
Molecular pathway of peretinoin. Peretinoin enhances the expression of multiple retinoid target genes. Through the retinoid target gene, peretinoin inhibits HCC proliferation, suppresses tumor growth and induces tumor apoptosis. In addition to enhancing retinoid target gene expression, peretinoin induces changes in the expression levels of a variety of genes involved in hepatocarcinogenesis, such as those related to Wnt signaling, IGF signaling, interferon, mTOR, and cell cycle regulation. Therefore, peretinoin modulates multiple signaling cascades involved in carcinogenesis, either directly or indirectly. HCC, hepatocellular carcinoma; IGF, insulin-like growth factor; mTOR, mammalian target of rapamycin; VEGF, vascular endothelial growth factor; PDGF, platelet-derived growth factor. * cytochrome P450, family 26, subfamily B, polypeptide 1, insulin-like growth factor binding protein 6, regulatory factor X-associated ankyrin-containing protein, putative lymphocyte G0/G1 switch gene, retinol binding protein 1, retinol binding protein 4, retinoic acid induced 3, transglutaminase 2, CCAAT/enhancer binding protein (C/EBP). ** junctional adhesion molecule 3, V-myc myelocytomatosis viral oncogene homolog, Src-like-adaptor, cell division cycle 2, G1 to S and G2 to M, BCL2-associated athanogene, chemokine (C-C motif) receptor 9, pre-B-cell leukemia transcription factor 1. ^§^ FK506 binding protein 12-rapamycin associated protein 1. ^¶^ Jumonji domain containing 3, jumping translocation breakpoint, protein kinase, AMP-activated, alpha 2 catalytic subunit. ^ƒ^ guanylate binding protein 1, interferon-inducible, 67kDa, interferon-induced protein 44, chemokine (C-X-C motif) ligand 9.

It is well known that abnormalities in the genes regulating Wnt signaling, IGF signaling, interferon, mTOR, and the cell cycle have been indicated to play a crucial role in the development of HCC [34,35]. Gene expression profiles in the liver identified candidate drug-response genes among the genes which exhibited changes in expression before and after peretinoin administration. These genes included Wnt signal-related, IGF signal-related, interferon-related, mTOR-related, and cell cycle-related genes known to be involved in the process of carcinogenesis of hepatocellular carcinoma, in addition to the retinoid-related genes which are the targets of peretinoin, suggesting that peretinoin directly or indirectly regulate many signal transduction systems in the carcinogenic process. Furthermore, with a hierarchical cluster analysis showing the feasibility of differentiating within-2-year-recurrence and non-recurrence groups, a comparative analysis of the two groups was performed. The results showed that, in the non-recurrence group, differentiation of hepatocytes and expression of the genes involved in tumor suppression were accelerated, while expression of the genes involved in promotion of liver fibrosis and lipidation, as well as the genes which can become markers for the stem cells of hepatic cancer was reduced. Changes in the expression of these genes may not reflect the direct action of peretinoin, but they were assumed to become eventual candidates for the genes related to drug efficacy. Furthermore, genes with a potential to distinguish the two doses were extracted by a comparison of the two dose groups of 300 mg and 600 mg of peretinoin. These genes included retinoid-related genes as well as genes that play a significant role in hepatic cancer and hepatic cirrhosis. The exact modes of action are not certain. Through these mechanisms, peretinoin suppresses HCC cell proliferation, and therefore could prevent HCC recurrence by modulating multiple signaling cascades involved in carcinogenesis, either directly or indirectly (Figure 1) [18,20].

The Wnt/β-catenin carcinogenesis pathway is frequently activated in HCC associated with hepatitis virus, especially HCV. As peretinoin has been shown to upregulate genes associated with the negative regulation of Wnt/β-catenin signaling, studies have investigated the chemopreventive activities of peretinoin in HCV- and HBV-associated HCCs [18,36]. In contrast, the β-catenin pathway is not activated in metabolic syndrome associated HCCs. Rather, carcinogenic mediators include insulin, lipid peroxidation and oxidative stress induced by free radicals, all of which stimulate cellular proliferation, activate hepatic progenitor cells and induce p53 mutations and epigenetic aberrations [37,38]. The non-activation of the Wnt/β-catenin pathway in metabolic syndrome associated HCCs suggested that peretinoin may not be effective as a chemopreventive agent in these patients. However, peretinoin also downregulates the expression of genes related to inflammation [18]. In a rat model, peretinoin targeted platelet-derived growth factor (PDGF) signaling, preventing hepatic fibrosis, steatosis and HCC development [20] and suggesting that peretinoin may be useful in the chemoprevention of metabolic syndrome related HCCs. It was reported that peretinoin activates the autophagy pathway by increasing Atg16L1 expression to prevent the progression of HCC in NAFLD mouse model [39]. Indeed, a recent preclinical study in obese and diabetic db/db mice with DEN-induced HCC showed that peretinoin significantly reduced the incidence of obesity related HCC [29]. Thus, peretinoin may be useful in preventing metabolic syndrome related HCC in humans, a hypothesis requiring testing in clinical trials.

### 2.3. Pharmacokinetics and Metabolism

Administration of single doses (300, 600 and 900 mg) of peretinoin to human subjects confirmed dose proportionality, with the lipid form of peretinoin reaching saturation at high doses. The route of drug administration is per oral. At all dose levels, urinary excretion of peretinoin was not observed [40]. Results of 24-week repeated-dose administration (150 mg × 2/day, 300 mg × 2/day, and 450 mg × 2/day) confirmed that peretinoin and its lipid form reached and maintained steady state concentrations in plasma for up to 24 weeks. Assessments of the elimination phase showed that peretinoin concentrations near the quantitation limit were present in some subjects after completion of administration at each dose. However, the level was lower than 1% of the Cmax after a single dose at each dose level. In most subjects, the plasma concentrations of peretinoin and peretinoin lipids were lower after completion of administration than the quantitation limit at 12 weeks. These results suggested that peretinoin does not accumulate in plasma, and that the steady state can be maintained for up to 24 weeks after administration. Moreover, food intake had no effect on plasma peretinoin concentration, as these concentrations did not differ in subjects administered a single of 300 mg peretinoin in the presence or absence of food [40]. A study of its clinical pharmacology showed dose-dependent increases in plasma peretinoin concentrations following 8-week repeated administration at doses of 300 mg/day and 600 mg/day. Peretinoin concentrations in the liver were below the lower quantitation limit (0.050 μg/g) in all six subjects administered 300 mg/day peretinoin and in four of the six subjects administered 600 mg/day peretinoin. The other two subjects administered 600 mg/day peretinoin had plasma peretinoin concentrations of 0.052 μg/g and 0.059 μg/g, respectively. Repeated-dose administration for up to 96 weeks showed no evidence of drug accumulation in the liver [18]

### 2.4. Clinical Efficacy

#### 2.4.1. Phase I Study (Study NIK-333-01 Conducted in Japan)

This study examined the safety and tolerability of a single dose of peretinoin (300, 600, or 900 mg) as well as 48 week repeated doses (300, 600, or 900 mg/day, twice daily) in patients who had undergone treatment for HCC. The administration method (pre- or post-prandial) was assessed to determine the effects of food intake, and the pharmacokinetics of peretinoin were investigated following single-dose and 24-week repeated-dose administration. Of the nine subjects receiving repeated doses of 900 mg/day peretinoin, four showed increases in blood pressure and required study discontinuation; these blood pressure increases were resolved by treatment with anti-hypertensive agents. In all cases of blood pressure increased, a causal relationship with the study drug was not ruled out. The 900 mg/day dose was therefore considered poorly tolerated. Steady state concentrations of free and lipid-associated peretinoin were maintained for up to 24 weeks with no indication of accumulation. The results of this study indicated that the maximum clinical dose was 600 mg/day, twice daily, and postprandial administration was selected because it was associated with good compliance. As increases in blood pressure can be controlled by treatment with anti-hypertensive agents, phase II clinical studies were considered justified [40,41].

#### 2.4.2. Clinical Pharmacology Study (Study NIK-333-02 Conducted in Japan)

This randomized parallel group open label study conducted in Japan focused on the clinical pharmacology of peretinoin. Peretinoin (300 or 600 mg/day, twice daily) was administered repeatedly for 8 weeks under open-label conditions to patients after curative therapy for HCV-positive HCC. Changes in gene expression profiles in the liver and in the peripheral blood before and after treatment and drug concentrations in the liver, plasma, and urine were compared in the 300 and 500 mg/day groups (step I). Then, peretinoin (600 mg/day, twice daily) was administered to all subjects for an additional 88 weeks (total treatment, 96 weeks) to investigate the safety of long-term repeated administration of peretinoin, focusing on the vascular system and changes over time in plasma drug concentration (step II). In addition, plasma TGF-α levels were measured as an exploratory predictive marker protein. Analyses, cluster analyses, and by-group predictions (comparing patients administered 300 and 600 mg/day, twice daily, peretinoin and patients with and without tumor recurrence within 2 years) were performed on the hepatic gene expression profiles before and after treatment with the study drug. From this gene expression profile, peretinoin responsive genes and genes related to HCC recurrence could be distinguished. The details have been discussed in the earlier section on pharmacodynamics. The pharmacokinetic results of this study were in agreement with those of the earlier NIK-333-01 study, as described above. No increase in plasma TGF-α level was observed at any time point in any subject, indicating that TGF-α is unsuitable as a predictive marker protein. Safety analysis showed that blood pressure tended to increase. These increases, however, were controlled by administration of anti-hypertensive agents, with no progression to arteriosclerosis. The mechanism of the blood pressure increased caused by the study drug remains unknown at present. There were no changes in blood pressure-associated parameters that could cause blood pressure to increase [18].

#### 2.4.3. Phase II/III Study (Study NIK-333-03 Conducted in Japan, Confirmatory Trial)

This placebo-controlled, multicenter, randomized, double-blind, phase II/III study included Child-Pugh A and B patients in Japan who had undergone curative therapy for HCV related HCC. Patients were administered peretinoin at two dose levels (300 or 600 mg/day, twice daily) or placebo for 96 weeks to evaluate the safety and efficacy of peretinoin, with recurrence free survival being the primary endpoint. Dose response was also investigated in this trial. Of 401 subjects randomized 1:1:1 to placebo, 300 mg/day peretinoin, and 600 mg/day peretinoin, 377 (94.0%) were evaluable for efficacy analysis, including 127, 126, and 124 in the above three groups, respectively. Recurrence-free survival did not differ significantly between the peretinoin groups and the placebo group (*p* = 0.434). However, there was a significant difference (*p* = 0.023) in the dose-response relationship analysis, as efficacy begins to increase at 600 mg/day. Secondary analysis found that, although the overall hazard ratio (HR) for risk of HCC recurrence/death in the 600 mg/day group was not significant, the HR during the second year of treatment was significant (HR 0.267, 95% confidence interval [CI] 0.074–0.961). These results suggested that twice daily administration of 600 mg/day peretinoin was clinically effective in preventing the recurrence of HCC [42].

Safety analysis showed that the overall rates of adverse events (AEs) in the placebo, 300 mg/day peretinoin, and 600 mg/day peretinoin groups were 97.7%, 96.9% and 97.7%, respectively. Adverse events that showed dose-dependent increases included ascites, diarrhea, abdominal discomfort, stomatitis, cheilitis, cystitis, urinary tract infection, back pain, osteoarthritis, hypertension, headache, dizziness, dermatitis, onychoclasis, the presence of urinary albumin and protein, and increased blood pressure. Most AEs were mild or moderate and remitted or disappeared, demonstrating that they were largely reversible and self-limiting. Ascites and arterial hypertension have not been previously reported as AEs of retinoids and hence appear to be specific to peretinoin. Of the patients in the placebo, 300 mg/day peretinoin, and 600 mg/day peretinoin groups, 4.7%, 6.9% and 15.9%, respectively, experienced AEs necessitating a discontinuation of study drug. This incidence increased significantly as the dosage of peretinoin increased (*p* = 0.02). AEs that necessitated study drug discontinuation and with a ≥1% higher incidence in the peretinoin groups than in the placebo group included anemia, headache, renal impairment, peripheral edema, edema, onychoclasis and increased transaminases. These findings suggested that 600 mg/day peretinoin was a reasonable clinical dose for suppression of recurrence of HBV-positive and HCV-positive HCC. The safety findings also indicated that peretinoin was well tolerated.

The original NIK-333-03 study did not evaluate overall survival among the study groups because the follow-up period is relatively short (median 2.5 years). Patients in the original study were therefore followed up for a median 4.9 years (maximum, 6.5 years) [43]. Dropout rates were minimal; four of 133 patients in the placebo group, three of 134 in the 300 mg/day peretinoin group, and two of 134 in the 600 mg/day peretinoin group. The 5-year cumulative overall survival rates in these three groups were 64.3%, 56.8%, and 73.9%, respectively, with no significant differences among these groups. However, in the subset of patients with Child-Pugh A liver score, the 5-year overall survival was significantly higher in the 600 mg/day peretinoin group than in the placebo group (hazard ratio [HR] 0.575, 95% CI 0.341–0.967, *p* = 0.0347). Subgroup analysis also showed that, compared with patients in the placebo group, the risks of recurrence in Child Pugh A patients (HR 0.60, 95% CI 0.41–0.89) and patients with HCC <2 cm (HR 0.41, 95% CI 0.23–0.73) were significantly lower in those administered 600 mg/day peretinoin.

Taken together, these results indicated that administration of 600 mg/day peretinoin to Child-Pugh A patients following curative treatment of HCV related HCC can significantly improve long-term survival. This is important for designing subsequent phase III trials.

#### 2.4.4. Ongoing Trials

##### Phase III study (Study NIK-333-05 conducted in Japan, Confirmatory Trial) (ClinicalTrials.gov identifier NCT01640808)

This placebo-controlled, multicenter, randomized, double-blind phase III registration trial in Japan was planned to enroll 600 patients. Patients were randomized to 600 mg/day peretinoin or placebo until HCC recurrence of HCC or death, or until completion or discontinuation of the entire study. The aim of this study is to evaluate the safety and efficacy of peretinoin for patients confirmed to have achieved complete remission of HCV-positive HCC, with the time from registration to recurrence or death (recurrence free survival) being the primary endpoint. Extrapolating from the long term outcomes of the earlier NIK-333-03 study, patients are restricted to those with Child-Pugh A liver function. Interim analysis of safety and efficacy will be performed. Recruitment has been completed and the study results are to be determined. In the study, 180 subjects (99 in the placebo group, 81 in the peretinoin group) used DAAs. While DAAs was coadministered with peretinoin, 133 adverse events occurred in 69 subjects in the placebo group and 124 in 56 subjects in the NIK-333 group, with no clinically significant problems. However, concomitant therapy with other antiviral agents induced hepatic dysfunction as an adverse drug reaction.

##### Phase III study (Study K-333-06 conducted in Japan, Exploratory Trial)

In this placebo-controlled, multicenter, randomized, double-blind, phase III study, patients who have achieved complete remission of HBV-associated HCC were randomized to 600 mg/day peretinoin or placebo twice daily for up to 4 years (range, 2.5–4 years). Safety and efficacy will be evaluated, with the time from registration to recurrence or death (recurrence free survival) being the primary endpoint. Recruitment has been completed and the study results are to be determined. In the study, although at least 90% of all patients used nucleic acid analogues concomitantly, there seems to be no clinically significant problems.

##### K-333-3.01AS

This study is being performed in Asian countries outside Japan to determine the ability of peretinoin to inhibit the recurrence of HCC in patients with complete remission of HBV and HCV associated HCC. Countries included are South Korea, Taiwan and Singapore, in which the primary etiology of HCC is HBV related, unlike in Japan, where the primary etiology is HCV related. The study protocol is similar to that of the large NIK-333-05 study in Japan, except that subjects can be positive for either HBV or HCV, but not for both. The study was planned to enroll 600 subjects. Recruitment has been completed and the study results are to be determined.

### 2.5. Safety and Tolerability

Table 2 shows AEs of ≥3% higher incidence in the peretinoin than in the placebo group in clinical studies conducted in Japan of patients with complete remission of HCV-positive HCC (Study NIK-333-03), and Table 3 shows severe adverse events (SAEs) of ≥1% higher incidence in the peretinoin than in the placebo group in the same clinical studies. The common AE profiles of peretinoin are similar to those of other retinoids, including vitamin A [44,45,46,47]. AEs apparently specific to peretinoin include ascites, albuminuria and arterial hypertension. Although these AEs are reversible and easily controlled, it is important to monitor for them, especially in subjects with renal impairment. Although peretinoin has not been used in Child-Pugh C patients, the incidence of SAEs was higher in Child-Pugh B than in Child-Pugh A patients [42]. Because peretinoin was found to be teratogenic in preclinical studies, it should not be administered to women with reproductive potential. Rather, peretinoin should be administered to these patients only if the expected therapeutic benefits outweigh the possible risks associated with treatment. Also, because peretinoin was shown to be excreted into breast milk, nursing mothers should discontinue breast feeding during administration of the drug. There is no post-marketing surveillance as peretinoin has not yet been approved for general clinical use.

## 3. Discussion and Future Perspectives

Secondary chemoprevention of HCC as adjuvant treatment is important, as the rates of HCC recurrence after initial curative therapy, including resection or local ablative therapy such as percutaneous ethanol injection or radiofrequency ablation, are high due to remaining underlying liver disease such as cirrhosis or metabolic syndrome [25,48]. Liver transplantation would be ideal, but is not readily available to treat HCC. The ideal drug for secondary chemoprevention of HCC after curative treatment should not only be effective but show long time safety, as secondary chemoprevention of HCC is lifetime issue. Currently, peretinoin, an orally available synthetic retinoid, is the only potential drug that is well into clinical development for secondary chemoprevention of HCC [21,49]. Peretinoin, a synthetic acyclic retinoid, has been shown to affect the expression of specific genes related to hepatocyte differentiation and tumor suppression, as well genes promoting liver fibrosis and steatosis and those encoding HCC stem cell markers, thereby inducing differentiation and apoptosis of hepatic stem cells and suppressing neoplastic clonal formation [18,20,29,50]. It inhibits multiple cellular signaling pathways linked to aberrant cell proliferation and to anti-apoptosis.

Peretinoin is thought to prevent HCC recurrence by inhibiting oncogenesis from precancerous lesions in the liver or by inhibiting the growth of occult hepatic cancer caused by various liver diseases [19,44,49]. Peretinoin has been suggested to especially prevent metachronous multicentric recurrence of HCC [18]. A phase II clinical trial found that recurrence free survival was enhanced only in HCV-positive HCC subjects treated with 600 mg/day peretinoin twice daily for at least 2 years [42]. 377 patients with curatively treated HCV-related HCC in phase III trial of peretinoin showed a lower trend of HCC recurrence for the study period (HR 0.73), and also up to two years after the study (HR 0.27). A longer overall survival in patients treated with with a higher dose of peretinoin compared to untreated controls was reported in a follow-up survey. The outcomes of two large phase III trials, in patients with HCV- and HBV-positive HCC, are awaited, as are the outcomes of another ongoing large scaled phase III study in Asian patients after complete remission of HBV- and HCV-positive HCC (Study K-333-3.01AS). Even if these phase III trials yield positive results, only a select group of patients may be expected to benefit from adjuvant peretinoin. A long-term evaluation of this population found that survival benefits were limited to the subgroup of patients with Child-Pugh A liver function. Moreover, its effects may be marginal and require long term continuous treatment. Patients must take four large capsules, each containing 75 mg peretinoin, twice daily, either indefinitely or until HCC recurrence. Therefore long term safety of peretinoin is of concerns. Of the adverse events reported to date, albuminuria and arterial hypertension are important, as their incidence in the peretinoin groups was over 15% and was dose dependent. Because most subjects had underlying liver cirrhosis, long term albuminuria and arterial hypertension would have negative effects on kidney function, requiring long-term monitoring for albuminuria and arterial hypertension. Most SAEs occurred in older subjects and those with Child-Pugh B liver function. These patients are more likely to have HCC and their kidneys more susceptible to the adverse effects of peretinoin than younger, Child-Pugh A patients [42,43].

The main risk factors for HCC worldwide are chronic HBV and HCV infection. The development of highly effective DAAs for HCV and new effective treatment strategies for HBV, are expected to significantly reduce the rates of HBV and HCV infections over the next 5 years thereby reducing the risks of HCC. NAFLD related fibrosis and cirrhosis are becoming important causes of HCC. Peretinoin has been well studied in patients with HCV and HBV related HCC. Although no clinical study to date has assessed the effects of peretinoin in patients with NAFLD/obesity related HCCs, pre-clinical studies have shown that peretinoin can inhibit DEN-induced liver tumorigenesis in obese and diabetic mice, suggesting that peretinoin may be also effective in preventing recurrence of NAFLD/obesity related HCC [21,39]. Combined treatment of adjuvant agents such as immune checkpoint inhibitors (ICIs) or Heparanase inhibitor PI-88 with the universal efficacy can be further considered to achieve enhanced efficacy and long term safety for the secondary pharmarcotherapy of HCC [51,52,53,54,55].

## 4. Conclusions

Peretinoin, a synthetic oral ACR discovered in 1980, has been specifically developed for secondary chemoprevention in patients who had undergone curative surgical or local ablative treatment for HCC. Clinical trials on the tolerability and efficacy of peretinoin indicated that twice daily administration of 600 mg/day peretinoin would be effective in preventing HCC recurrence and that peretinoin was safe and tolerable. Its long-term safety and marginal efficacy with hepatitis C virus- or hepatitis B virus-associated HCC for its clinical use as adjuvant therapy in patients is of concern. Subsequent placebo-controlled phase III clinical trials are underway. The outcome of these phase III trials will determine whether peretinoin is approved for clinical use. Therefore, the dose of peretinoin (2 × 600 mg/day) might be considered high as for the anticancer drug under clinical development. To exert more effective and satisfactory outcomes of ACR to enable HCC chemoprevention, the combination therapy of ACR with more potent anticancer agents would be considered [49,56].

## Figures and Tables

**Table 1:** Peretinoin clinical studies.

Protocol	Phase	Endpoints	Subjects	Design	Number of Subjects
**Muto et al.** **(1996)**			Post-curative treatment of HCC (HBV, HCV, NBNC)	Randomized, double blinded, placebo control	89
**NIK-333-01** **(Japan)**	I	PK, safety	Post-curative treatment of HCC (HBV, HCV, B+C, NBNC)	Open label	33
**NIK-333-02** **(Japan)**	I	Gene expression, PK, safety	Post-curative treatment of HCC (HCV)	Randomized, parallel group, open label	12
**NIK-333-03** **(Japan)**	II/III	Efficacy, safety	Post-curative treatment of HCC (HCV)	Randomized, double blinded, parallel group, placebo control	401
**K-333-1.01EU** **(UK)**	I	PK, safety	Japanese and Caucasian males volunteer	Open, crossover, single administration/ Double-blind, randomized, parallel-group, placebo-controlled, repeated administration	41
**K-333-07** **(JAPAN)**	I	PK, Safety on QT/QTc interval	Healthy male volunteer	double-blind, randomized, placebo- and positive-control	56
**NIK-333-05** **(Japan)**	III (ongoing)	Efficacy, safety	Post-curative treatment of HCC (HCV)	Randomized, double blinded, parallel group, placebo control	600
**K-333-06** **(Japan)**	III (ongoing)	Efficacy, safety	Post-curative treatment of HCC (HBV)	Randomized, double blinded, parallel group, placebo control	100
**K-333-3.01A** **(KR/TW/SG)**	III (ongoing)	Efficacy, safety	Post-curative treatment of HCC (HBV or HCV)	Randomized, double blinded, parallel group, placebo control	600

HCC, hepatocellular carcinoma—HBV, hepatitis B virus—HCV, Hepatitis C virus—NBNC, non-B non-C—PK, pharmacokinetics—EU, European Union—UK, United Kingdom—KR, Republic of Korea—TW, Taiwan—SG, Singapore.

**Table 2:** Adverse events and adverse drug reactions of incidence ≥3% higher in the peretinoin groups than in the placebo group (specific adverse events).

SOC (MedDRA ver.12.0) PT (MedDRA ver.12.0)	Adverse Events						Adverse Drug Reactions					
	Placebo Group		300 mg/day Peretinoin Group		600 mg/day Peretinoin Group		Placebo Group		300 mg/day Peretinoin Group		600 mg/day Peretinoin Group	
	*N* = 129		*N* = 131		*N* = 132		*N* = 129		*N* = 131		*N* = 132	
	*n*	%	*n*	%	*n*	%	*n*	%	*n*	%	*n*	%
Gastrointestinal disorders												
Ascites *	8	6.2	15	11.5	21	15.9	0	0.0	3	2.3	9	6.8
Diarrhea *	7	5.4	10	7.6	16	12.1	1	0.8	1	0.8	2	1.5
Varices esophageal *	11	8.5	15	11.5	13	9.8	2	1.6	1	0.8	0	0.0
Constipation	5	3.9	10	7.6	8	6.1	1	0.8	0	0.0	0	0.0
Abdominal discomfort	2	1.6	4	3.1	8	6.1	1	0.8	0	0.0	2	1.5
Stomatitis	2	1.6	5	3.8	7	5.3	0	0.0	1	0.8	2	1.5
Cheilitis	0	0.0	1	0.8	7	5.3	0	0.0	0	0.0	2	1.5
Gastritis	2	1.6	7	5.3	4	3.0	0	0.0	1	0.8	1	0.8
Reflux esophagitis	0	0.0	5	3.8	3	2.3	0	0.0	1	0.8	0	0.0
Infections and infestations												
Nasopharyngitis *	46	35.7	57	43.5	50	37.9	3	2.3	1	0.8	1	0.8
Cystitis	4	3.1	6	4.6	9	6.8	0	0.0	0	0.0	0	0.0
Urinary tract infection	0	0.0	6	4.6	8	6.1	0	0.0	1	0.8	2	1.5
Musculoskeletal and connective tissue disorders												
Back pain *	10	7.8	11	8.4	17	12.9	2	1.6	1	0.8	1	0.8
Osteoarthritis	0	0.0	1	0.8	4	3.0	0	0.0	0	0.0	0	0.0
Blood and lymphatic system disorders												
Anemia	2	1.6	1	0.8	7	5.3	2	1.6	0	0.0	2	1.5
Iron deficiency anemia	0	0.0	0	0.0	4	3.0	0	0.0	0	0.0	1	0.8
Vascular disorders												
Hypertension	4	3.1	10	7.6	12	9.1	4	3.1	10	7.6	11	8.3
Respiratory, thoracic and mediastinal disorders												
Pleural effusion	1	0.8	0	0.0	5	3.8	0	0.0	0	0.0	1	0.8
Injury, poisoning and procedural complication												
Lumbar vertebral fracture	1	0.8	1	0.8	5	3.8	0	0.0	0	0.0	2	1.5
Nervous system disorders												
Headache *	11	8.5	15	11.5	17	12.9	4	3.1	9	6.9	11	8.3
Dizziness	4	3.1	5	3.8	9	6.8	0	0.0	1	0.8	1	0.8
General disorders and administration site conditions												
Edema peripheral*	11	8.5	11	8.4	16	12.1	1	0.8	3	2.3	8	6.1
Pyrexia	8	6.2	13	9.9	12	9.1	1	0.8	1	0.8	2	1.5
Edema	4	3.1	3	2.3	10	7.6	1	0.8	0	0.0	3	2.3
Thirst	2	1.6	1	0.8	4	3.0	0	0.0	1	0.8	4	3.0
Chest discomfort	0	0.0	4	3.1	1	0.8	0	0.0	1	0.8	0	0.0
Metabolism and nutrition disorders												
Hypokalemia	1	0.8	5	3.8	3	2.3	0	0.0	2	1.5	0	0.0
Skin and subcutaneous tissue disorders												
Pruritus	9	7.0	12	9.2	11	8.3	0	0.0	6	4.6	6	4.5
Nail disorder	2	1,6	7	5.3	4	3.0	2	1.6	6	4.6	4	3.0
Dermatitis	0	0.0	2	1.5	4	3.0	0	0.0	1	0.8	3	2.3
Onychoclasis	0	0.0	1	0.8	4	3.0	0	0.0	1	0.8	4	3.0
Investigation												
Albumin urine present *	8	6.2	14	10.7	29	22.0	5	3.9	11	8.4	26	19.7
Blood pressure increased *	19	14.7	20	15.3	26	19.7	16	12.4	16	12.2	21	15.9
Transaminases increased *	15	11.6	10	7.6	23	17.4	7	5.4	5	3.8	11	8.3
Protein urine present	0	0.0	2	1.5	8	6.1	0	0.0	0	0.0	8	6.1
Blood urine present	3	2.3	7	5.3	5	3.8	1	0.8	3	2.3	3	2.3

*n* = Number of relevant subjects, *N* = Total number of subjects, * Specific adverse events with an incidence ≥ 10% higher in the 300 or 600 mg peretinoin group than in the placebo group.

**Table 3:** Serious adverse events of incidence ≥1% higher in either peretinoin group than in the placebo group, and rates of hepatic neoplasm malignant recurrent.

SOC (MedDRA ver.12.0) PT (MedDRA ver.12.0)	Adverse Events						Adverse Drug Reactions					
	Placebo Group		300 mg/day Peretinoin Group		600 mg/day Peretinoin Group		Placebo Group		300 mg/day Peretinoin Group		600 mg/day Peretinoin Group	
	*N* = 129		*N* = 131		*N* = 132		*N* = 129		*N* = 131		*N* = 132	
	*n*	%	*n*	%	*n*	%	*n*	%	*n*	%	*n*	%
Gastrointestinal disorders												
Ascites	1	0.8	1	0.8	8	6.1	0	0.0	1	0.8	5	3.8
Varices esophageal	4	3.1	7	5.3	5	3.8	0	0.0	0	0.0	0	0.0
Gastric ulcer hemorrhage	0	0.0	0	0.0	3	2.3	0	0.0	0	0.0	1	0.8
Gastric varices	0	0.0	2	1.5	1	0.8	0	0.0	0	0.0	0	0.0
Infections and infestations												
Urinary tract infection	0	0.0	3	2.3	0	0.0	0	0.0	0	0.0	0	0.0
Hepatobiliary disorders												
Hepatic failure	0	0.0	2	1.5	1	0.8	0	0.0	1	0.8	1	0.8
Cardiac disorders												
Cardio-respiratory arrest	0	0.0	0	0.0	2	1.5	0	0.0	0	0.0	2	1.5
Nervous system disorders												
Hepatic encephalopathy	0	0.0	2	1.5	2	1.5	0	0.0	1	0.8	0	0.0
General disorders and administration site conditions												
Pyrexia	0	0.0	1	0.8	2	1.5	0	0.0	0	0.0	0	0.0
Neoplasms benign, malignant and unspecified (incl. cysts and polyps)												
Gastric cancer stage 0	0	0.0	1	0.8	3	2.3	0	0.0	1	0.8	0	0.0

*n* = Number of relevant subjects, *N* = Total number of subjects.
